# Intra-Individual Reproducibility of Automated Abdominal Organ Segmentation—Performance of TotalSegmentator Compared to Human Readers and an Independent nnU-Net Model

**DOI:** 10.1007/s10278-024-01265-w

**Published:** 2024-09-18

**Authors:** Lorraine Abel, Jakob Wasserthal, Manfred T. Meyer, Jan Vosshenrich, Shan Yang, Ricardo Donners, Markus Obmann, Daniel Boll, Elmar Merkle, Hanns-Christian Breit, Martin Segeroth

**Affiliations:** Department of Radiology, University Hospital Basel, Petersgraben 4, 4031 Basel, Switzerland

**Keywords:** Reproducibility, CT, Segmentation, Neural networks

## Abstract

The purpose of this study is to assess segmentation reproducibility of artificial intelligence-based algorithm, TotalSegmentator, across 34 anatomical structures using multiphasic abdominal CT scans comparing unenhanced, arterial, and portal venous phases in the same patients. A total of 1252 multiphasic abdominal CT scans acquired at our institution between January 1, 2012, and December 31, 2022, were retrospectively included. TotalSegmentator was used to derive volumetric measurements of 34 abdominal organs and structures from the total of 3756 CT series. Reproducibility was evaluated across three contrast phases per CT and compared to two human readers and an independent nnU-Net trained on the BTCV dataset. Relative deviation in segmented volumes and absolute volume deviations (AVD) were reported. Volume deviation within 5% was considered reproducible. Thus, non-inferiority testing was conducted using a 5% margin. Twenty-nine out of 34 structures had volume deviations within 5% and were considered reproducible. Volume deviations for the adrenal glands, gallbladder, spleen, and duodenum were above 5%. Highest reproducibility was observed for bones (− 0.58% [95% CI: − 0.58, − 0.57]) and muscles (− 0.33% [− 0.35, − 0.32]). Among abdominal organs, volume deviation was 1.67% (1.60, 1.74). TotalSegmentator outperformed the reproducibility of the nnU-Net trained on the BTCV dataset with an AVD of 6.50% (6.41, 6.59) vs. 10.03% (9.86, 10.20; *p* < 0.0001), most notably in cases with pathologic findings. Similarly, TotalSegmentator’s AVD between different contrast phases was superior compared to the interreader AVD for the same contrast phase (*p* = 0.036). TotalSegmentator demonstrated high intra-individual reproducibility for most abdominal structures in multiphasic abdominal CT scans. Although reproducibility was lower in pathologic cases, it outperforms both human readers and a nnU-Net trained on the BTCV dataset.

**Supplementary Information:**

The online version contains supplementary material available at 10.1007/s10278-024-01265-w.

## Introduction

In recent years, various artificial intelligence-based segmentation algorithms have been proposed [1–7]. Given their enormous potential clinical impact, especially for longitudinal analysis, segmentation reproducibility is of utmost importance [8–10]. Accurate and robust image segmentation algorithms are helpful tools for providing quantitative results. They are of great value in surgical planning and radiation therapy [1] and may be leveraged to quantify tumor burden [11]. Furthermore, they are also an important step towards computer-aided diagnosis [12]. Due to their possible implications in medical diagnostic and treatment, the reproducibility of the segmentation algorithms is of fundamental importance [13]. Several technical improvements of algorithms were suggested to increase reproducibility [8, 14]. The TotalSegmentator by Wasserthal et al. [3], which was developed at our institution, achieved a strong segmentation performance with an nnU-Net [15, 16] by increasing the size and diversity of the training dataset, which was annotated by humans. Especially, the publicly available training dataset of TotalSegmentator included different contrast phases (unenhanced, arterial, portal venous, late phase, and others) and examinations with aberrations like tumor, vascular pathologies, trauma, inflammation, bleeding, and others [3].

Paschali et al. defined the reproducibility of a segmentation algorithm as the arising performance gap when introducing adversarial examples to the test data [17]. These adversarial examples are often perturbed images and indistinguishable from the original to the human eye [14]. When multiple segmentation models are compared, reproducibility can be assessed on the same images [18]; however, when reproducibility is evaluated in a single segmentation algorithm, data augmentation is performed so far [13]. Nevertheless, this data augmentation does not resemble a real world variance in data presented to segmentation algorithms that are used in a clinical environment. In a clinical context, CT images vary substantially upon the administration of contrast agents or in the presence of pathologies. Nevertheless, these situations demand an equal performance of the automated segmentation.

The aim of this study was therefore to evaluate TotalSegmentator [3] in a patient cohort with clinically suspected active gastrointestinal hemorrhage, thus including challenging pathological characteristics and compare the performance against the reproducibility of a nnU-Net trained on the BTCV dataset and inter-human reader variation. Each examination requires a triphasic CT protocol, allowing to evaluate segmentation reproducibility across consecutive scans in the same patient. We hypothesized that the segmented volume of the abdominal organs, muscles, and bones using TotalSegmentator should be highly reproducible with minimal volume deviations across different contrast phases. Furthermore, reproducibility should likely decrease for CT examinations with pathologic findings, as those cases are less frequent in the training dataset.

## Materials and Methods

The study was carried out according to the principles of the Declaration of Helsinki and was approved by the local ethics committee (Req-2023–00446). Written informed consent was waived due to the retrospective nature of the study.

### Study Design and Study Sample

All patients who underwent triphasic CT abdomen-pelvis examinations (including unenhanced, arterial, and portal venous acquisitions at 1–1.5 mm slice thickness) for clinically suspected active gastrointestinal bleeding at our institution between January 1, 2012, and December 31, 2022, were retrospectively included. Examinations with missing of one or more contrast phases, incomplete image acquisition of the abdomen and pelvis, and missing CT slices or corrupted data were excluded. Also, examinations that were part of the initial TotalSegmentator training data were excluded. Anonymized data were stored on the Nora Imaging Platform [19], where evaluation and manual segmentation was performed.

### CT Imaging

CT examinations were acquired on a Somatom Definition Force, Somatom Definition Flash, or a Somatom Definition AS + scanner (Siemens Healthineers, Germany). The imaging protocol represented our standard-of-care for suspected gastrointestinal bleeding and included unenhanced, arterial, and portal venous scans at a tube voltage of 100–120 kV. Following the unenhanced scan, 1–1.5 ml/kg body weight of 370 mg I/ml iopromide (Ultravist® 370, Bayer Pharma) was administered intravenously. Using bolus tracking technique, arterial phase images were acquired 18 s after reaching 100 HU in the descending aorta at the level of the celiac trunk. Portal venous phase images were obtained 70 s after reaching the scan initiation threshold.

### Abdominal Fluid Identification

One radiology resident with 3 years of experience, supervised by a board-certified radiologist with 7 years of experience, identified all cases with pathological fluid, including gastrointestinal, intraperitoneal, retroperitoneal, extraperitoneal, or intraparenchymal hemorrhage, as well as ascites based on the images and the respective radiology reports. These cases will be referred to as “fluid” cases and all other cases as “non-fluid” cases.

### Automated, Semi-Automated, and Manual Segmentation

Multiple segmentations were performed for comparison (Supplement Fig. 1). First, 34 abdominal organs and structures as listed and grouped in Table 1 and Fig. 2 were automatically segmented using TotalSegmentator v2.0.1 [3] on all three contrast phases of each CT examination. Deviations in volume measurements were used to assess the reproducibility between contrast phases.

**Fig. 1:**
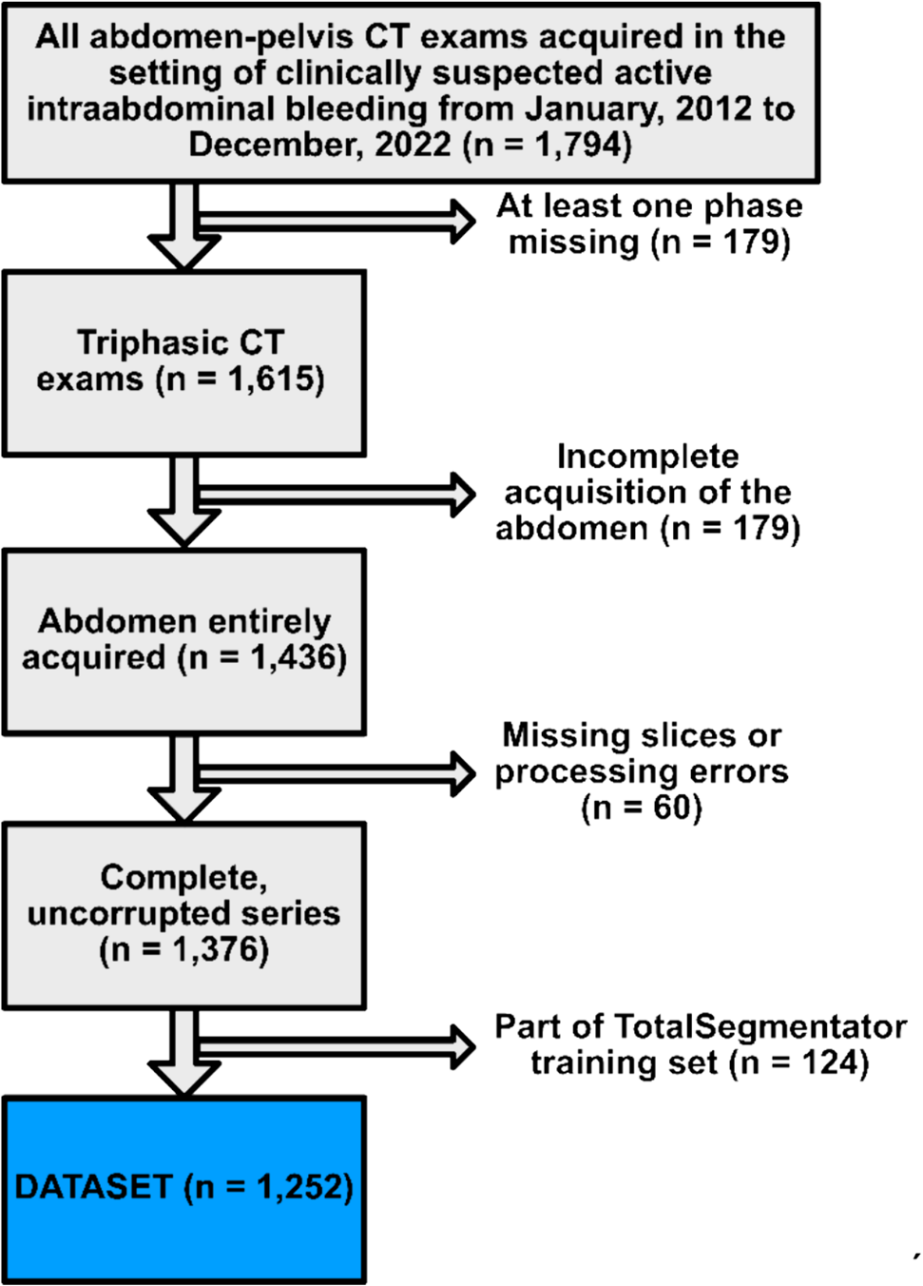
Flowchart of the study sample

**Table 1:** Pseudomedian volumes of anatomic structures from automated TotalSegmentator segmentations on different CT contrast phases and *p* values for pairwise non-inferior testing between contrast phases

Structure	Contrast phases			Non-inferiority test					
	Portal venous	Arterial	Unenhanced	UE vs. PV		ART vs. PV		UE vs. ART	
				LM	UM	LM	UM	LM	UM
Parenchymal abdominal organs									
Liver	1625 (1596, 1654)	1627 (1598, 1656)	1616 (1587, 1646)	< 0.00001	< 0.00001	< 0.00001	< 0.00001	< 0.00001	< 0.00001
Spleen	218 (209, 226)	208 (200, 217)	214 (205, 222)	< 0.0001	< 0.00001	0.2633*	< 0.00001	< 0.00001	< 0.00001
Pancreas	63 (62, 65)	62 (61, 63)	62 (61, 64)	< 0.00001	< 0.00001	< 0.00001	< 0.00001	< 0.00001	< 0.00001
Kidney (left)	147 (145, 150)	145 (142, 148)	142 (139, 145)	< 0.00001	< 0.00001	< 0.00001	< 0.00001	< 0.00001	< 0.00001
Kidney (right)	146 (143, 149)	144 (141, 147)	140 (137, 143)	< 0.00001	< 0.00001	< 0.00001	< 0.00001	< 0.00001	< 0.00001
Adrenal gland (left)	5 (5, 5)	5 (5, 5)	4 (4, 5)	0.9995*	< 0.00001	< 0.00001	< 0.00001	< 0.01*	< 0.00001
Adrenal gland (right)	4 (4, 4)	4 (4, 4)	4 (3, 4)	1.0000*	< 0.00001	< 0.00001	< 0.00001	1.0000*	< 0.00001
Hollow abdominal organs									
Gallbladder	35 (33, 37)	34 (32, 36)	33 (31, 34)	0.2879*	< 0.00001	< 0.0001	< 0.00001	< 0.00001	< 0.00001
Urinary bladder	179 (171, 188)	174 (166, 183)	174 (165, 182)	< 0.00001	< 0.00001	< 0.00001	< 0.00001	< 0.00001	< 0.00001
Stomach	351 (336, 367)	352 (337, 368)	350 (335, 365)	< 0.00001	< 0.00001	< 0.00001	< 0.00001	< 0.00001	< 0.00001
Duodenum	59 (57, 60)	58 (57, 59)	56 (55, 58)	< 0.001*	< 0.00001	< 0.00001	< 0.00001	< 0.00001	< 0.00001
Small bowel	1050 (1027, 1074)	1051 (1028, 1075)	1045 (1022, 1069)	< 0.00001	< 0.00001	< 0.00001	< 0.00001	< 0.00001	< 0.00001
Colon	857 (836, 879)	853 (832, 875)	858 (836, 880)	< 0.00001	< 0.00001	< 0.00001	< 0.00001	< 0.00001	< 0.00001
Bones									
Vertebra T12	48 (47, 48)	48 (47, 48)	48 (48, 49)	< 0.00001	< 0.00001	< 0.00001	< 0.00001	< 0.00001	< 0.00001
Vertebra L1	54 (54, 55)	54 (54, 55)	55 (54, 55)	< 0.00001	< 0.00001	< 0.00001	< 0.00001	< 0.00001	< 0.00001
Vertebra L2	60 (59, 60)	60 (59, 61)	60 (59, 61)	< 0.00001	< 0.00001	< 0.00001	< 0.00001	< 0.00001	< 0.00001
Vertebra L3	66 (65, 67)	67 (66, 67)	67 (66, 68)	< 0.00001	< 0.00001	< 0.00001	< 0.00001	< 0.00001	< 0.00001
Vertebra L4	67 (66, 67)	67 (66, 68)	67 (66, 68)	< 0.00001	< 0.00001	< 0.00001	< 0.00001	< 0.00001	< 0.00001
Vertebra L5	65 (65, 66)	66 (65, 67)	66 (65, 67)	< 0.00001	< 0.00001	< 0.00001	< 0.00001	< 0.00001	< 0.00001
Hip (left)	390 (385, 394)	389 (385, 394)	392 (388, 396)	< 0.00001	< 0.00001	< 0.00001	< 0.00001	< 0.00001	< 0.00001
Hip (right)	389 (385, 393)	389 (385, 394)	392 (387, 396)	< 0.00001	< 0.00001	< 0.00001	< 0.00001	< 0.00001	< 0.00001
Sacrum	228 (225, 230)	228 (226, 230)	230 (228, 232)	< 0.00001	< 0.00001	< 0.00001	< 0.00001	< 0.00001	< 0.00001
Femur (left)	168 (166, 171)	166 (163, 168)	167 (164, 169)	< 0.00001	< 0.00001	< 0.00001	< 0.00001	< 0.00001	< 0.00001
Femur (right)	167 (165, 170)	165 (162, 167)	166 (164, 169)	< 0.00001	< 0.00001	< 0.00001	< 0.00001	< 0.00001	< 0.00001
Muscles									
Autochthon (left)	387 (381, 394)	385 (379, 391)	381 (375, 387)	< 0.00001	< 0.00001	< 0.00001	< 0.00001	< 0.00001	< 0.00001
Autochthon (right)	383 (376, 389)	380 (374, 386)	375 (369, 381)	< 0.00001	< 0.00001	< 0.00001	< 0.00001	< 0.00001	< 0.00001
Iliopsoas (left)	253 (247, 258)	252 (247, 258)	257 (251, 262)	< 0.00001	< 0.00001	< 0.00001	< 0.00001	< 0.00001	< 0.00001
Iliopsoas (right)	247 (241, 252)	246 (241, 251)	250 (245, 256)	< 0.00001	< 0.00001	< 0.00001	< 0.00001	< 0.00001	< 0.00001
Gluteus medius (left)	232 (228, 235)	233 (229, 236)	234 (230, 238)	< 0.00001	< 0.00001	< 0.00001	< 0.00001	< 0.00001	< 0.00001
Gluteus medius (right)	227 (223, 231)	227 (223, 231)	228 (225, 232)	< 0.00001	< 0.00001	< 0.00001	< 0.00001	< 0.00001	< 0.00001
Gluteus minimus (left)	58 (58, 59)	58 (57, 59)	58 (57, 59)	< 0.00001	< 0.00001	< 0.00001	< 0.00001	< 0.00001	< 0.00001
Gluteus minimus (right)	64 (63, 64)	63 (62, 64)	64 (63, 65)	< 0.00001	< 0.00001	< 0.00001	< 0.00001	< 0.00001	< 0.00001
Gluteus maximus (left)	443 (434, 451)	435 (427, 444)	441 (432, 449)	< 0.00001	< 0.00001	< 0.00001	< 0.00001	< 0.00001	< 0.00001
Gluteus maximus (right)	456 (448, 465)	449 (440, 458)	455 (446, 464)	< 0.00001	< 0.00001	< 0.00001	< 0.00001	< 0.00001	< 0.00001

Data are pseudomedian volumes in ml with associated 95% confidence intervals. For non-inferior testing, a Wilcoxon signed rank test with a 5% margin for the lower and upper bound was used, as this was considered reproducible. *p* values indicated by a * are not significant after Bonferroni correction. *UE*, unenhanced; *ART*, arterial; *PV*, portal venous; *LM*, lower margin; *UM*, upper margin

**Fig. 2:**
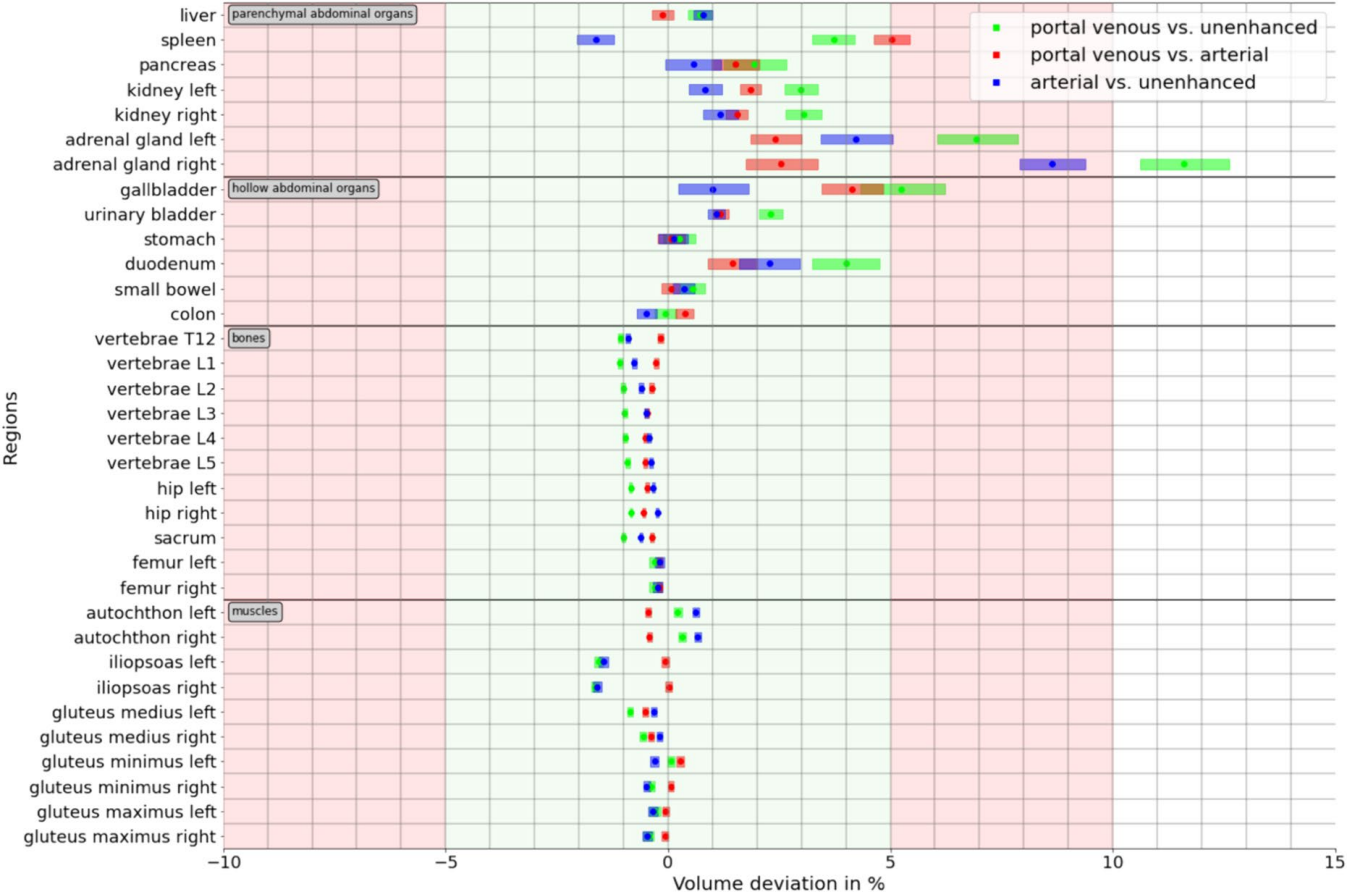
Percent volume deviations of automated segmentations by TotalSegmentator between different CT contrast phases stratified by anatomic structures. Data are given as pseudomedians (dots) with associated 95% CIs (shaded rectangles) for pairwise comparisons of CT contrast phases using color coding, including portal venous vs. unenhanced images (green), portal venous vs. arterial phase (red), and arterial vs. unenhanced (blue)

Second, liver, spleen, pancreas, kidneys, adrenal glands, gallbladder, and stomach were automatically segmented on all three contrast phases of each examination using a nnU-Net trained on the Multi-Atlas Labeling Beyond the Cranial Vault—Workshop and Challenge dataset (https://www.synapse.org/#!Synapse:syn3193805/wiki/217780) (“BTCV dataset”) acquired at Vanderbilt University Medical Center [20]. These abdominal structures were selected as these are the only abdominal organs that are contained with segmentations in the dataset. The training of the nnU-Net trained on the BTCV dataset was described previously [3]. The absolute volume deviations between contrast phases were calculated and compared to absolute volume deviations of TotalSegmentator’s segmentations to compare reproducibility. The nnU-Net trained on the BTCV dataset was selected for comparison as the BTCV dataset is one of the most used open source training datasets with multiple anatomical segmentations on CT data.

Third, two human readers, one radiology resident with 1 year of experience (reader 1) and one board-certified radiologist with 7 years of experience (reader 2), manually segmented the liver on portal venous phase images of 20 randomly selected cases (10 fluid and 10 non-fluid cases). Absolute volume deviations between the two readers in the same contrast phase were calculated, as well as DICE score and compared to absolute volume deviations and DICE score of TotalSegmentator between any two different contrast phases to TotalSegmentator’s performance compared to inter-human reproducibility.

Forth, one human reader, a radiology resident with 3 years of experience, manually corrected TotalSegmentator’s automated segmentations of the liver, spleen, both kidneys, both adrenal glands, and pancreas in all three contrast phases in 30 randomly selected non-fluid cases. These organs were selected as greater volume deviations than expected were observed in this subgroup of organs. This correction step was performed to evaluate whether there was an actual deviation in volumes between contrast phases due to the administration of contrast agent, or if deviations were caused by segmentation errors of TotalSegmentator.

### Metrics

To examine the similarity of segmentations, the volume deviation (VD) was calculated pairwise between contrast phases for each organ and structure

1 $$\text{VD}=\frac{x-y}{x}.$$

Comparisons were performed as follows: *y*: unenhanced vs. *x*: portal venous, *y*: arterial vs. *x*: portal venous, and *y*: unenhanced vs. *x*: arterial. In contrast to volume similarity, VD is capable to indicate a change in terms of an over- or under-segmentation as defined in [21, 22]. A DICE score or a normalized surface distance (NSD) score would not indicate the direction of change and was not possible due to imperfect alignment between the contrast phases. An average Hausdorff distance might rank a segmentation containing more errors better than a segmentation with less errors because the denominator changes with the number of voxels in the segmentation. Thus, as stated previously [23], the traditional average Hausdorff distance should be used with caution for rankings and quality assessment of segmentations. Consequently, VD was thought to most accurately represent small changes in segmentations between contrast phases and used as metric of segmentation reproducibility. A VD closer to zero indicates a higher reproducibility and a VD within 5% was considered reproducible.

Thus, to compare between patient groups (non-fluid vs. fluid) and segmentation methods (TotalSegmentator, nnU-Net trained on the BTCV dataset, human, manually corrected TotalSegmentator), we used the absolute value of the volume deviation (absolute volume deviation (AVD))

2 $$\text{AVD}=\frac{\left|x-y\right|}{x}=|\text{VD}|,$$

as suggested by Babalola et al. [24]. A lower AVD value suggests higher equality between segmentations and thus higher overall reproducibility. Nevertheless, a theoretical perfect AVD would be achieved by ignoring the underlying image and returning the same results. However, this also holds for the DICE and NSD score and would be inconsistent with the accurate results of TotalSegmentator [3] and would be noticed when compared to human readers.

When two readers segmented in the same contrast phase, the calculation of the DICE score is adequate as there is perfect alignment between the images; however, these had to be compared to DICE scores of TotalSegmentator between two images with possible imperfect alignment.

### Statistical Analysis

Normal distribution of the volumes of the respective segmented anatomical structures was rejected for most abdominal organs and structures by using the Kolmogorov–Smirnov test. Continuous variables were reported as pseudomedian and associated 95% confidence intervals (CI) using an underlying signed rank distribution. Whenever anatomical structures were grouped, the average was taken across all examinations and segmentations and contrast phases, respectively. Comparison between two groups was performed using the Wilcoxon signed rank test. Hypothesis testing was two-tailed. The underlying statistical method for non-inferior testing was a Wilcoxon signed rank test with a 5% margin for the lower and upper bound, as this was considered reproducible. Bonferroni-correction was performed. The significance level was set below the required level of *p* < 0.000245 to 0.0001. All statistical analyses were performed using Python 3.8.8.

## Results

### Study Sample

Out of 1794 triphasic abdominal CT examinations performed between January 1, 2012, and December 31, 2022, 542 had to be excluded as per the predefined criteria (Fig. 1). The final study sample consisted of 1252 CT examinations from 1079 patients (pseudomedian age: 73 [95% CI: 72, 74], 500/1252 (40%) female). Twenty-one percent (257/1252) of cases demonstrated a pathologic amount of intra-abdominal fluid (“fluid cases”). The remaining 79% (995/1252) of cases were “non-fluid cases.” Out of the 257 fluid cases, 83% (214/257) had ascites, 23% (58/257) had an intra-abdominal bleeding, and 6% (15/257) had ascites and intra-abdominal bleeding.

### Reproducibility of TotalSegmentator Between Contrast Phases

In all 1252 included studies, TotalSegmentator successfully segmented 34 abdominal organs and structures automatically on all three contrast phases. A list of all structures is given in Table 1.

Overall VD between contrast phases using TotalSegmentator was − 0.12% (95% CI: − 0.14, − 0.11). The smallest deviation from zero was observed between arterial and portal venous phases (0.00% [− 0.03, 0.02]), followed by unenhanced vs. portal venous phase (− 0.16% [− 0.19, − 0.13]) and unenhanced vs. arterial phase (− 0.21% [− 0.23, − 0.18]).

Bones and muscles showed the smallest VD from zero with − 0.58% (− 0.58, − 0.57) and − 0.33% (− 0.35, − 0.32), respectively. Organs demonstrated a larger VD with an average of 1.67% (1.60, 1.74). Among these, the right adrenal gland showed the largest deviation (7.58% [7.08, 8.09]), and the colon the smallest deviation (− 0.04% [− 0.17, 0.09]). VD of the liver was 0.47% [0.33, 0.60], spleen 2.30% [2.01, 2.57], pancreas 1.32% [0.97, 1.68], left kidney 1.87% [1.69, 2.06], and right kidney 1.86% [1.67, 2.06], respectively. Details for all 34 organs and structures are summarized in Fig. 2 and Table 1.

Non-inferiority testing demonstrated that segmented volumes of all osseous and muscular structures varied less than 5% (Fig. 2) between any contrast phase (all *p* < 0.0001) when using TotalSegmentator for segmentations. Regarding parenchymal abdominal organs, segmentation volumes for both adrenal glands, spleen, gallbladder, and duodenum volumes varied by more than 5% between contrast phases and did not demonstrate non-inferiority. For all other abdominal organs, VD was less than 5% between contrast phases (Fig. 2, Table 1).

### Performance with Intra-Abdominal Fluid

Segmentation reproducibility with TotalSegmentator was superior in non-fluid cases for all contrast phases (AVD non-fluid 2.01% [1.98, 2.03] vs. AVD fluid 2.39% [2.33, 2.45]; *p* < 0.0001). Regarding non-fluid vs. fluid comparisons between individual contrast phases, AVD for arterial phase vs. portal venous phase was 1.72% (1.68, 1.76) vs. 1.98% (1.89, 2.07; *p* < 0.0001), AVD for unenhanced vs. arterial phase was 2.00% (1.96, 2.04) vs. 2.44% (2.33, 2.55; *p* < 0.0001), and AVD for unenhanced vs. portal venous phase was 2.31% (2.26, 2.35) vs. 2.79% (2.67, 2.91; *p* < 0.0001).

**Fig. 3:**
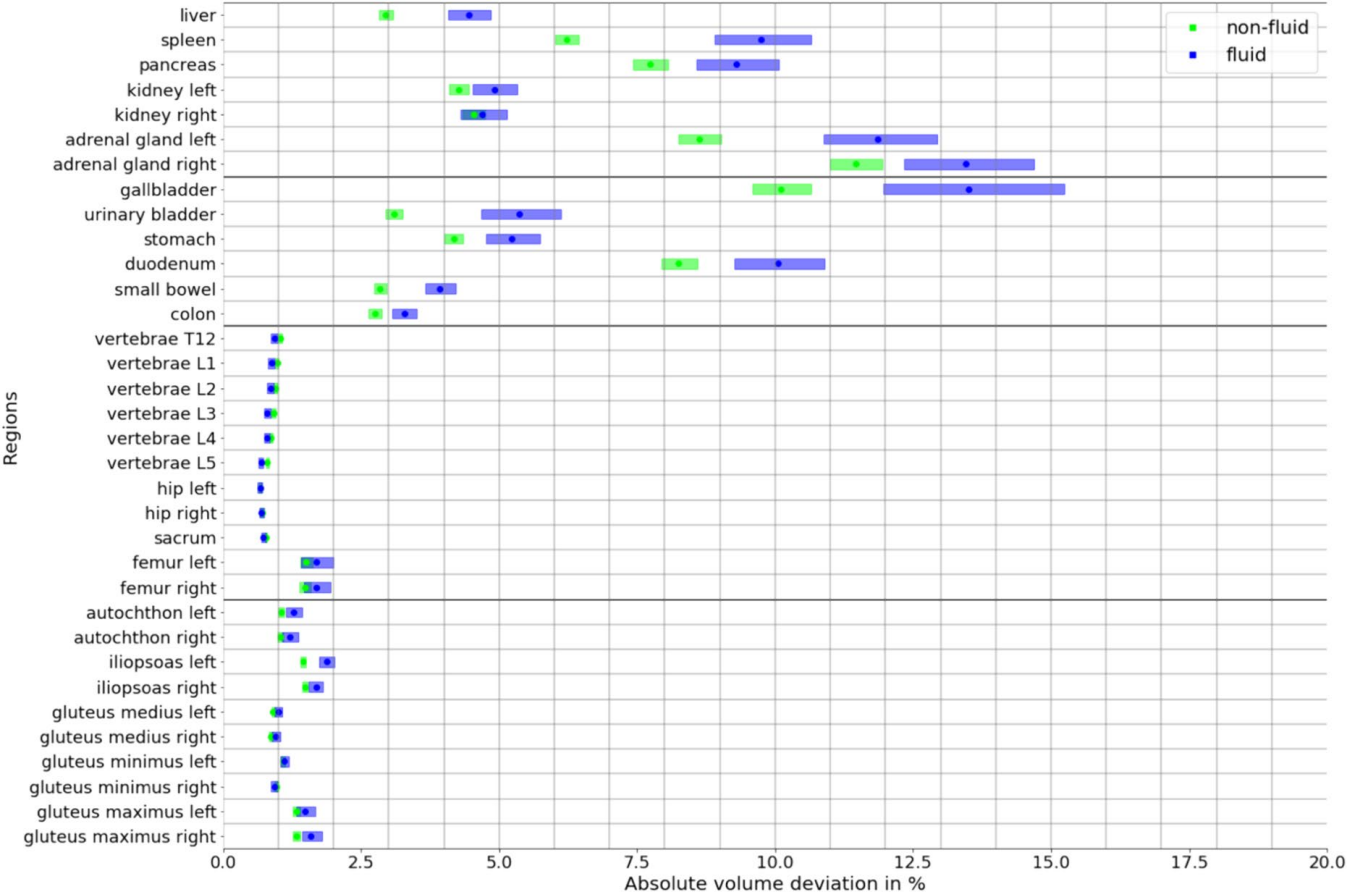
Comparison of percent absolute volume deviations of automated segmentations by TotalSegmentator in fluid (blue) and non-fluid cases (green), given as pseudomedians (dots) with associated 95% CIs (shaded rectangles)

For bones, the AVD over all contrast phases was 0.90% (0.89, 0.91) vs. 0.84% (0.83, 0.86; *p* < 0.0001). For muscles, the AVDs were 1.13% (1.12, 1.15) vs. 1.27% (1.24, 1.31; *p* < 0.0001). The parenchymal abdominal organs demonstrated the largest AVDs of all structures in both non-fluid (5.40% [5.33, 5.48]) and fluid cases (6.98% [6.79, 7.18]; *p* < 0.0001). Figure 3 illustrates the AVDs in fluid and non-fluid cases over all contrast phases for each segmented anatomical structure.

### Comparison with nnU-Net Trained on the BTCV Dataset and Interreader Reproducibility

**Fig. 4:**
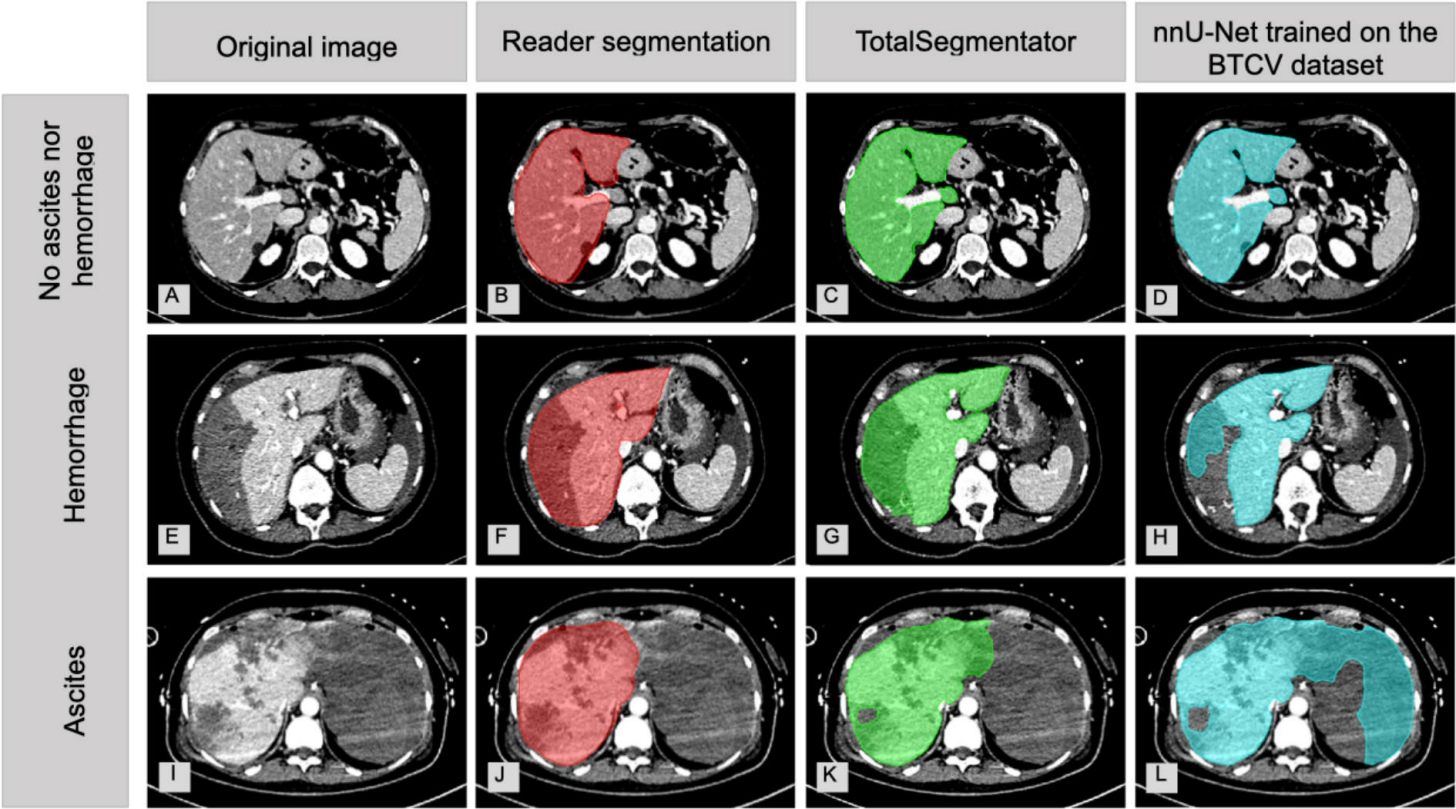
Side-by-side comparison of baseline CT images (A, E, I), manual human reader segmentations (B, F, J), automated TotalSegmentator segmentations (C, G, K), and automated segmentations using a nnU-Net trained on the BTCV dataset (D, H, L) in a non-fluid case (A–D), a case with liver hemorrhage (E–H), and a case with ascites (I–L)

**Fig. 5:**
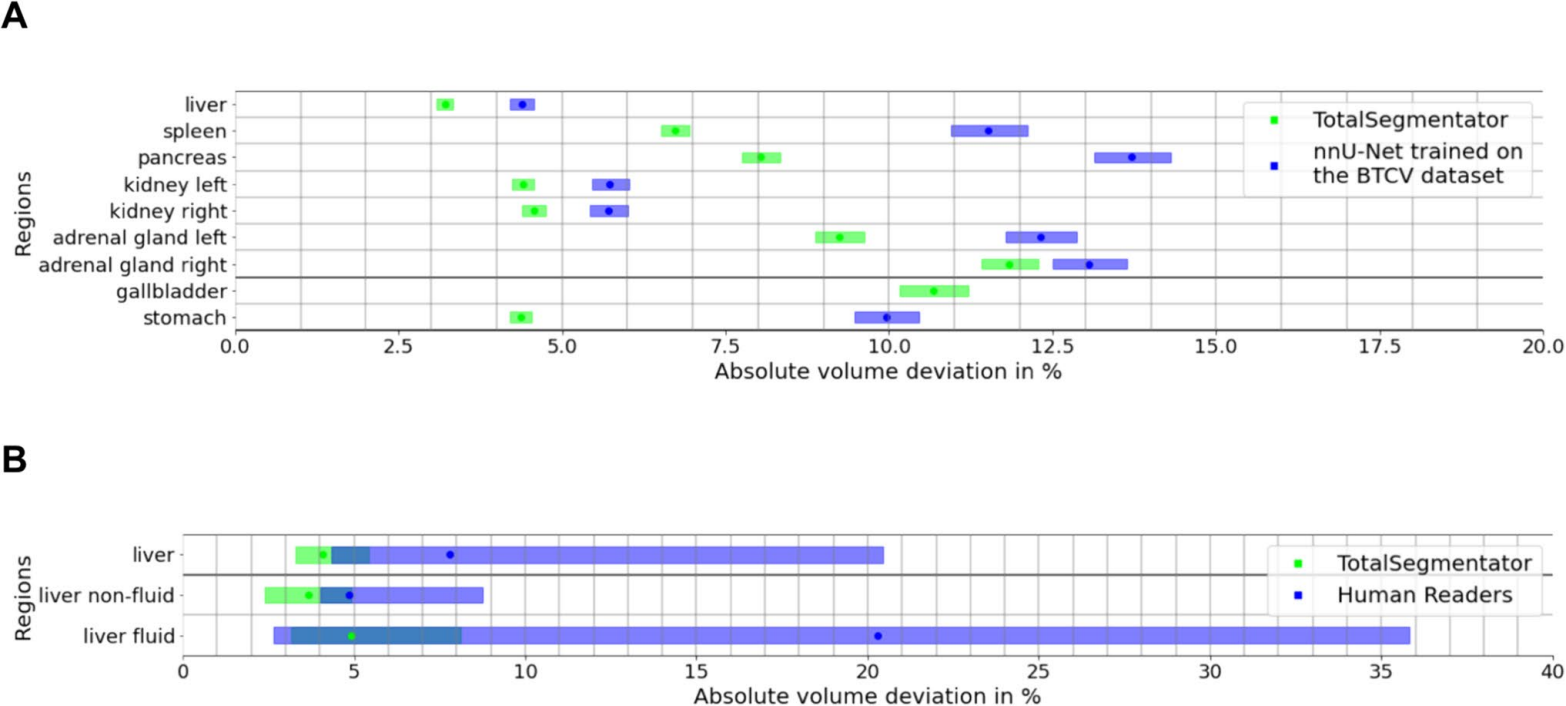
Comparison of percent absolute volume deviations between TotalSegmentator (green) and a nnU-Net trained on the BTCV dataset (blue) for nine abdominal organs (**A**) and between TotalSegmentator (green) and human readers (blue) in the liver overall, in fluid, and in non-fluid cases (**B**). Data are given as pseudomedians (dots) with associated 95% CIs (shaded rectangles)

The reproducibility of TotalSegmentator was superior compared to the nnU-Net trained on the BTCV dataset (Fig. 4). TotalSegmentator demonstrated a significant lower AVD of 6.50% (6.41, 6.59) compared to the nnU-Net trained on the BTCV dataset with an AVD of 10.03% (9.86, 10.20; *p* < 0.0001). In fluid cases, this difference was more pronounced with 7.93% (7.67, 8.19) vs. 16.21% (15.59, 16.87; *p* < 0.0001) for the nnU-Net trained on the BTCV dataset (Fig. 5A).

For human readers with manual segmentations, the AVD for the liver on portal venous phase images across 20 cases was 7.78% (4.35, 20.46), whereas TotalSegmentator’s AVD for the liver across all phases for the same 20 cases was lower with 4.10% (3.27, 5.42; *p* = 0.036). For non-fluid cases, human readers’ AVD was only slightly higher compared to that of TotalSegmentator with 4.84% (4.04, 8.75) vs. 3.69% (2.39, 4.93; *p* = 0.16). TotalSegmentator’s superiority in reproducibility was even more pronounced in absolute numbers in fluid cases with AVDs of 20.29% (2.66, 35.82) for the human readers vs. 4.91% (3.15, 8.11; *p* = 0.086) for TotalSegmentator, though this was not statistically significant (Fig. 5B). Regarding the DICE score, between the two human readers on the same contrast phase the DICE score amounted to 90% (84, 93), whereas the DICE score for TotalSegmentator between two phases was not significant higher (arterial vs. unenhanced: 94% (90, 95), *p* = 0.23; portal venous vs. arterial: 94% (92, 95), *p* = 0.19; portal venous vs. unenhanced: 92% (88, 94), *p* = 0.39).

### Effect of Contrast Agent Bolus Application on Parenchymal Organ Volume

**Fig. 6:**
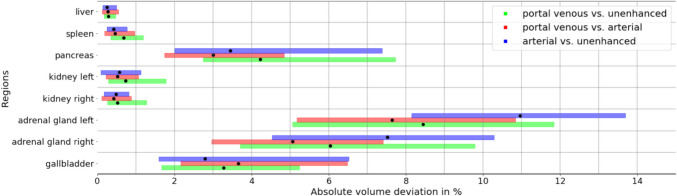
Differences in percent absolute volume deviations between initial automated and manually corrected TotalSegmentator segmentations, stratified as pairwise comparisons between contrast phases. Data are given as pseudomedians (dots) with associated 95% CIs (shaded rectangles)

Following contrast administration, there were notable increases in segmentation volumes of parenchymatous organs (Fig. 2), though visual inspection showed excellent segmentation results from TotalSegmentator in all three contrast phases, particularly for the liver and the spleen. Manual correction of TotalSegmentator segmentations was performed in 30 randomly selected cases and confirmed the previously noted increases in volume of parenchymatous organs following contrast administration. As demonstrated in Fig. 6, there was only a minor difference between the AVDs of manually corrected segmentations and automated segmentations by TotalSegmentator for the liver, spleen, and both kidneys on all three contrast phases. The other organs demonstrated higher AVDs following manual correction.

## Discussion

In this study, we analyzed the intra-individual reproducibility of automated segmentations by the segmentation algorithm TotalSegmentator through comparison of segmentation results across multiple contrast phases in abdominal CT scans. Triphasic CT examinations of the abdomen and pelvis provide three consecutive CT scans of the same patient within a short time span. Reproducibility is a key factor when investigating the validity, generalizability, and robustness of AI-based algorithms in radiology [25], addressing the inherent challenges of clinical datasets. This involves handling multiple contrast phases and accommodating both physiological and pathological patient anatomy. Indeed, the administration of contrast agents can alter CT attenuation differences between adjacent structures, potentially affecting the reproducibility of automated segmentation across contrast phases [5]. Furthermore, being publicly available for transparency purposes is essential for reproducibility [26, 27]. TotalSegmentator is an open-access whole body segmentation algorithm.

Regarding the reproducibility of TotalSegmentator, of the 34 analyzed abdominal structures 29 demonstrated a volume deviation below 5% between any of the three contrast phases, which was therefore considered as clinically reproducible. Among organs, reproducibility was best for the colon. Excellent reproducibility was also found for the liver, confirming the results from a previous investigation [2]. However, we observed a volume deviation above 5% for both adrenal glands, the gallbladder, the duodenum, and the spleen. The lower reproducibility in these organs can be explained by their small size and/or more complex shapes [28, 29] and in case of the spleen by the above described effect of contrast agent application. Here, a segmentation difference of a few voxels already leads to a large relative difference in volumes. This is in accordance with published literature, e.g., by Weston et al., who obtained a lower DICE score for the automated segmentation of adrenal glands compared to the liver with the same algorithm [7]. The lower reproducibility of duodenum and gallbladder segmentations can also be explained by inherent errors in the TotalSegmentator segmentation. Already in the initial publication of TotalSegmentator lower DICE scores were reported for these two anatomic structures [3].

We observed lower segmentation reproducibility of TotalSegmentator in cases with intra-abdominal fluid. A possible explanation may lie in reduced attenuation differences between organs and fluid compared to intra-abdominal adipose tissue. Indeed, typical liver attenuation values of 50–60 HU [30] are closer to those of hemorrhage (typically ranging from 35 to 100 HU) and ascites (ranging from − 10 HU to 15 HU), than to negative attenuation values of intra-abdominal and retroperitoneal fat. Dependent on the contrast phase, this may lead to more segmentation errors and lower reproducibility.

TotalSegmentator outperformed segmentations of an independent nnU-Net trained on the BTCV dataset both overall and in pathological cases for all nine analyzed structures. This is likely attributed to the training of TotalSegmentator, which was performed on a large dataset of 1204 clinically acquired CT exams in various contrast phases, whereas the BTCV dataset only included 30 CT exams in portal venous phase. Although the nnU-Net trained on the BTCV dataset was trained on much fewer data, the reproducibility for fluid cases was comparable to the human readers. Similarly, TotalSegmentator demonstrated superior performance across all contrast phases compered to two human readers regarding reproducibility, who performed segmentations of the liver in portal venous phase in cases with and without intra-abdominal fluid. This is in line with a previous work from Winkel et al. [2].

We noted a volume increase in parenchymatous organs after contrast administration despite visually excellent segmentation results in all three contrast phases, especially for the liver and the spleen. Despite manual segmentation correction in 30 randomly selected cases, volume increases persistent and were therefore thought to represent a physiologic phenomenon. A potential explanation might be the osmotically driven increase in blood flow after intravascular injection of approximately 200 ml combined contrast agent and saline solution-bolus. This would match previous studies, outlining decreases in spleen size under hypovolemic conditions [31], which increased with recovery [32]. This phenomenon could have influenced reproducibility of intra-individual segmentations across different contrast phases. A share of the observed VDs might therefore be attributed to physiological changes following contrast administration rather than segmentation errors of TotalSegmentator. Following this logic, the actual reproducibility of TotalSegmentator segmentations might be even better than the VDs we reported and may in part explain the lower reproducibility for the spleen.

In future research this deviation may be assessed in detail to give accurate volume estimations independent of the contrast phase. Furthermore, reproducibility can be assessed further comparing augmented data with real world data and development of algorithms for medical image perturbation may be performed. This may lead to more accurate image segmentations that can be used in the clinical setting and for development of computer-aided diagnosis tools.

The main limitation in our study is that Sørensen-Dice similarity coefficient for interphase reproducibility could not be used due to poor alignment of images between contrast phases, mostly due to patient motion. Secondly, this dataset was not curated and includes pathology and likely metal artifacts. This might have impaired the performance of TotalSegmentator. Thirdly, the intra-individual differences in segmentation volumes may be not solely attributed to TotalSegmentator, but in part also to the observed increases in the volume of parenchymatous organs after contrast administration. Despite these limitations, TotalSegmentator demonstrated a high reproducibility even in complex clinical cases.

## Conclusion

TotalSegmentator demonstrated high reproducibility for the majority of the assessed structures in multiphasic abdominal CT scans. TotalSegmentator outperforms a nnU-Net trained on the BTCV dataset and human readers on healthy cases as well as pathologic cases. Our results should encourage the testing of AI-based algorithms on reproducibility as an important criterion for clinical acceptability.

## Supplementary Information

Below is the link to the electronic supplementary material.

Supplementary file1 (DOCX 2709 KB)

## Abbreviations

AVD: Absolute volume deviation
VD: Volume deviation
