# Supplementary material for: Intra-Individual Reproducibility of Automated Abdominal Organ Segmentation—Performance of TotalSegmentator Compared to Human Readers and an Independent nnU-Net Model
Source: J Imaging Inform Med. 2024 Sep 18;38(3):1617–27. doi: 10.1007/s10278-024-01265-w (PMC12092311; doi:10.1007/s10278-024-01265-w)
Supplement: Supplementary file 1 — Supplementary file1 (DOCX 2709 KB) [file 10278_2024_1265_MOESM1_ESM.docx]

**SUPPLEMENT**


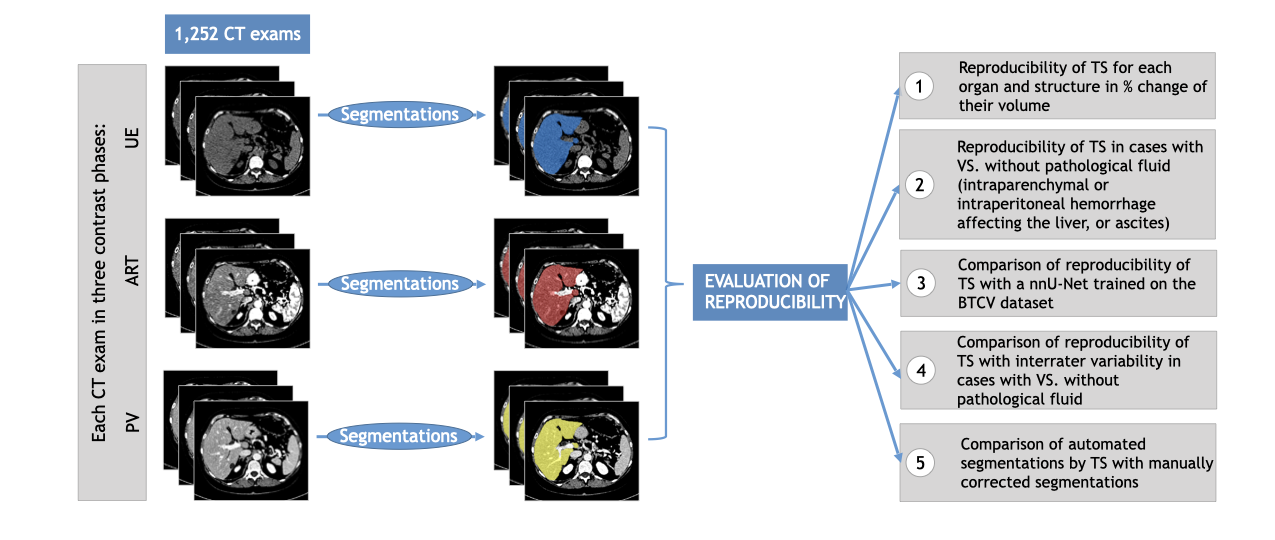


**Supplement Figure 1**: Processing of Image segmentation and evaluation. All three contrast (PV: portal venous, ART: arterial, UE: unenhanced) phases of the 1,252 CT examinations were segmented using TotalSegmentator (TS). Segmentation results were then used for evaluation of reproducibility and comparison with other segmentations.
